# Draft genome sequence of type strain HBR26^T^ and description of *Rhizobium aethiopicum* sp. nov.

**DOI:** 10.1186/s40793-017-0220-z

**Published:** 2017-01-26

**Authors:** Aregu Amsalu Aserse, Tanja Woyke, Nikos C. Kyrpides, William B. Whitman, Kristina Lindström

**Affiliations:** 1 Department of Environmental Sciences, University of Helsinki, Viikinkaari 2a, Helsinki, Finland; 2 DOE Joint Genome Institute, Walnut Creek, USA; 3 Department of Microbiology, University of Georgia, Biological Sciences Building, Athens, USA

**Keywords:** *Rhizobium aethiopicum*, Ethiopia, Common bean, Symbiotic, Genome, Average Nucleotide Identity

## Abstract

*Rhizobium aethiopicum* sp. nov. is a newly proposed species within the genus *Rhizobium*. This species includes six rhizobial strains; which were isolated from root nodules of the legume plant *Phaseolus vulgaris* growing in soils of Ethiopia. The species fixes nitrogen effectively in symbiosis with the host plant *P. vulgaris,* and is composed of aerobic, Gram-negative staining, rod-shaped bacteria. The genome of type strain HBR26^T^ of *R. aethiopicum* sp. nov. was one of the rhizobial genomes sequenced as a part of the DOE JGI 2014 Genomic Encyclopedia project designed for soil and plant-associated and newly described type strains. The genome sequence is arranged in 62 scaffolds and consists of 6,557,588 bp length, with a 61% G + C content and 6221 protein-coding and 86 RNAs genes. The genome of HBR26^T^ contains *repABC* genes (plasmid replication genes) homologous to the genes found in five different *Rhizobium etli* CFN42^T^ plasmids, suggesting that HBR26^T^ may have five additional replicons other than the chromosome. In the genome of HBR26^T^, the nodulation genes *nodB, nodC, nodS, nodI, nodJ* and *nodD* are located in the same module, and organized in a similar way as *nod* genes found in the genome of other known common bean-nodulating rhizobial species. *nodA* gene is found in a different scaffold, but it is also very similar to *nodA* genes of other bean-nodulating rhizobial strains. Though HBR26^T^ is distinct on the phylogenetic tree and based on ANI analysis (the highest value 90.2% ANI with CFN42^T^) from other bean-nodulating species, these *nod* genes and most nitrogen-fixing genes found in the genome of HBR26^T^ share high identity with the corresponding genes of known bean-nodulating rhizobial species (96–100% identity). This suggests that symbiotic genes might be shared between bean-nodulating rhizobia through horizontal gene transfer. *R. aethiopicum* sp. nov. was grouped into the genus *Rhizobium* but was distinct from all recognized species of that genus by phylogenetic analyses of combined sequences of the housekeeping genes *recA* and *glnII*. The closest reference type strains for HBR26^T^ were *R. etli* CFN42^T^ (94% similarity of the combined *recA* and *glnII* sequences) and *Rhizobium bangladeshense* BLR175^T^ (93%). Genomic ANI calculation based on protein-coding genes also revealed that the closest reference strains were *R. bangladeshense* BLR175^T^ and *R. etli* CFN42^T^ with ANI values 91.8 and 90.2%, respectively. Nevertheless, the ANI values between HBR26^T^ and BLR175^T^ or CFN42^T^ are far lower than the cutoff value of ANI (> = 96%) between strains in the same species, confirming that HBR26^T^ belongs to a novel species. Thus, on the basis of phylogenetic, comparative genomic analyses and ANI results, we formally propose the creation of *R. aethiopicum* sp. nov. with strain HBR26^T^ (=HAMBI 3550^T^=LMG 29711^T^) as the type strain. The genome assembly and annotation data is deposited in the DOE JGI portal and also available at European Nucleotide Archive under accession numbers FMAJ01000001-FMAJ01000062.

**Electronic supplementary material:**

The online version of this article (doi:10.1186/s40793-017-0220-z) contains supplementary material, which is available to authorized users.

## Introduction

Some bacteria are capable of forming a nitrogen-fixing symbiosis with various herbal and woody legumes. Some other bacterial species involve in nitrogen-fixation as free-living soil organisms [1]. Biological nitrogen fixation by root-nodule forming bacteria in symbiosis with legume plants play significant roles in agricultural systems. The symbiosis provides a nitrogen source for the legumes and consequently improve legume growth and agricultural productivity.

Common bean (*Phaseolus vulgaris*) (http://plants.usda.gov/core/profile?symbol=PHVU) is one of the best-known legume plants cultivated worldwide for food. It was originally domesticated in its Mesoamerican gene center, including Mexico, Colombia, Ecuador and northern Peru [2] and in the Andean center in the regions from Southern Peru to northern Argentina [3]. At present, it is widely cultivated in several parts of the tropical, sub-tropical and temperate agricultural systems [4] and used as a vital protein source mainly for low-income Latin Americans and Africans [5]. In Ethiopia, beans are commonly grown as a sole crop or intercropped with cereals, such as sorghum and maize, at altitudes between 1400 and 2000 m above sea level [6]. Bean plants make symbiotic associations promiscuously with several root-nodule forming nitrogen-fixing bacterial species commonly known as rhizobia. Studies thus far show that this legume forms symbiotic associations mainly with rhizobia belong to *Alphaproteobacteria*, such as *Rhizobium phaseoli*, *Rhizobium tropici* [7], *Rhizobium leguminosarum* [8]*,* *Rhizobium etli* [8], *Rhizobium giardinii*, *Rhizobium gallicum* [9]*,* *Rhizobium leucaenae* [10], *Rhizobium lusitanum* [11 *]*, *Rhizobium vallis* [12], *Rhizobium ecuadorense* [13]. *Rhizobium mesoamericanum* [14], *Rhizobium freirei* [15], *Rhizobium azibense* [16], *Rhizobium acidisoli* [17], *Ensifer meliloti* [18], *Ensifer fredii* [19], *Ensifer medicae* [20] and *Ensifer americanum* [21]. Rhizobial species belonging to *Betaproteobacteria*, such as *Burkholderia phymatum* [22] was also found capable of forming nodules on common bean plants.

16S rRNA gene sequence similarity and DNA–DNA hybridization techniques have been used as standard methods for describing new bacterial species. However, the 16S rRNA gene sequence divergence between closely related species is low and thus cannot differentiate closely related species found in the same genus [23–25]. The DDH technique was once considered as the gold standard method, and strains classified in the same species should have 70% or greater DDH relatedness among each other [26–29]. However, DDH results vary between different laboratories and this incurs inconsistent classification of the same species [30]. On the other hand, the multilocus sequence analysis method, using the sequences of several housekeeping protein coding genes, have been successfully used for species identification and delineation [24, 25, 31, 32]. The genome-wide ANI method, which was first proposed by Konstantinidis and Tiedje [33] has recently successfully been used for classification of various bacterial species [34, 35]. Depending on the methods used for ANI calculation or the nature of bacterial genome sequences, 95 or 96.5% ANI value [34, 35] corresponds to the classical 70% DNA–DNA relatedness cutoff value for strains of the same species. The advancement of sequencing techniques and its falling price have made genomic data for many bacterial species available for comparison [36]. Consequently, the ANI is becoming the method of choice in current bacterial taxonomic studies.

In our previous study, we isolated a group of rhizobial bacteria from nodules of common bean growing in the soils of Ethiopia. These bacteria formed a unique branch that was distinct from recognized species of the genus *Rhizobium* in phylogenetic trees constructed based on MLSA [24]. In order to compare strains using genome-wide ANI with reference genomes and to describe this group as a new *Rhizobium* species, the representative strain *Rhizobium* sp. HBR26 (hereafter *Rhizobium aethiopicum* sp. nov. HBR26 ^T^) was selected for sequencing. This project was a part of the DOE JGI 2014 Genomic Encyclopedia of Type Strains, Phase III, the genomes of soil and plant-associated and newly described type strains sequencing program [37]. In this study, we present classification and general features of *R. aethiopicum* sp. nov. including the description of the genome sequence and annotation of the type strain HBR26 ^T^.

## Organism information

### Classification and features

**Fig. 1:**
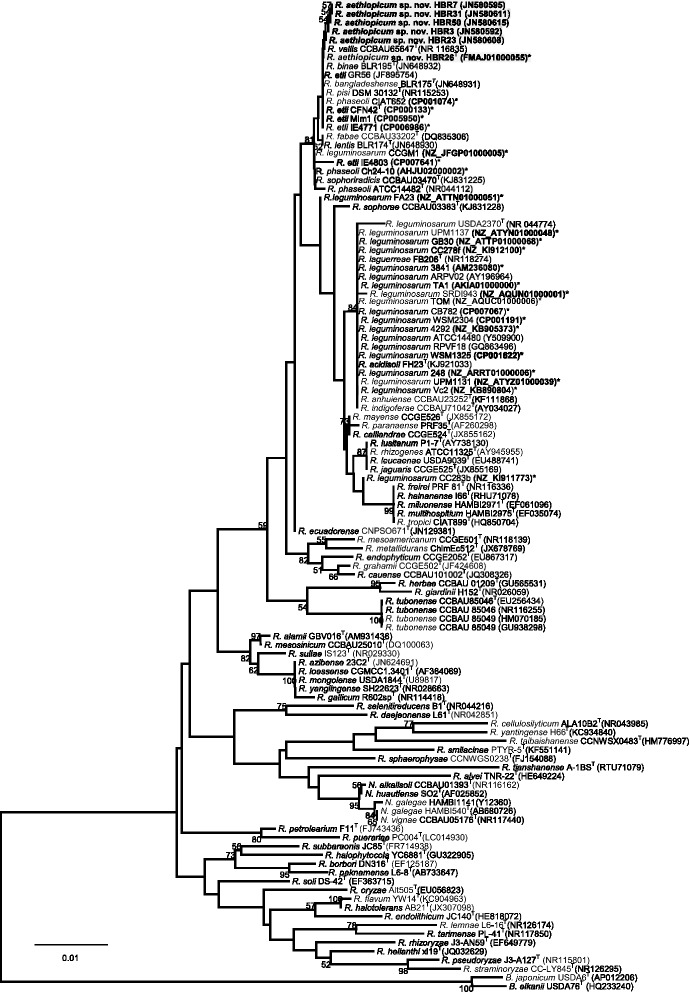
Neighbor-Joining phylogenetic tree reconstructed based partial 16S rRNA gene sequences (801 bp), showing the relationships between *Rhizobium aethiopicum* sp. nov (bold and highlighted) and recognized species of the genus *Rhizobium*. The tree was computed using the Kimura 2-parameter model using MEGA version 7. The rate variation among sites was modeled with a gamma distribution (shape parameter = 4). Bootstrap values (1000 replicates) are shown at the branching points. Reference type strains are indicated with superscript ‘T’. Bar, % estimated substitutions. GenBank accession numbers of the sequences are indicated in parentheses next to strains codes. The accession numbers of whole genome sequenced strains are indicated with bold*. Abbreviations: B, *Bradyrhizobium*; R, *Rhizobium*; N, *Neorhizobium*; sp., species

**Fig. 2:**
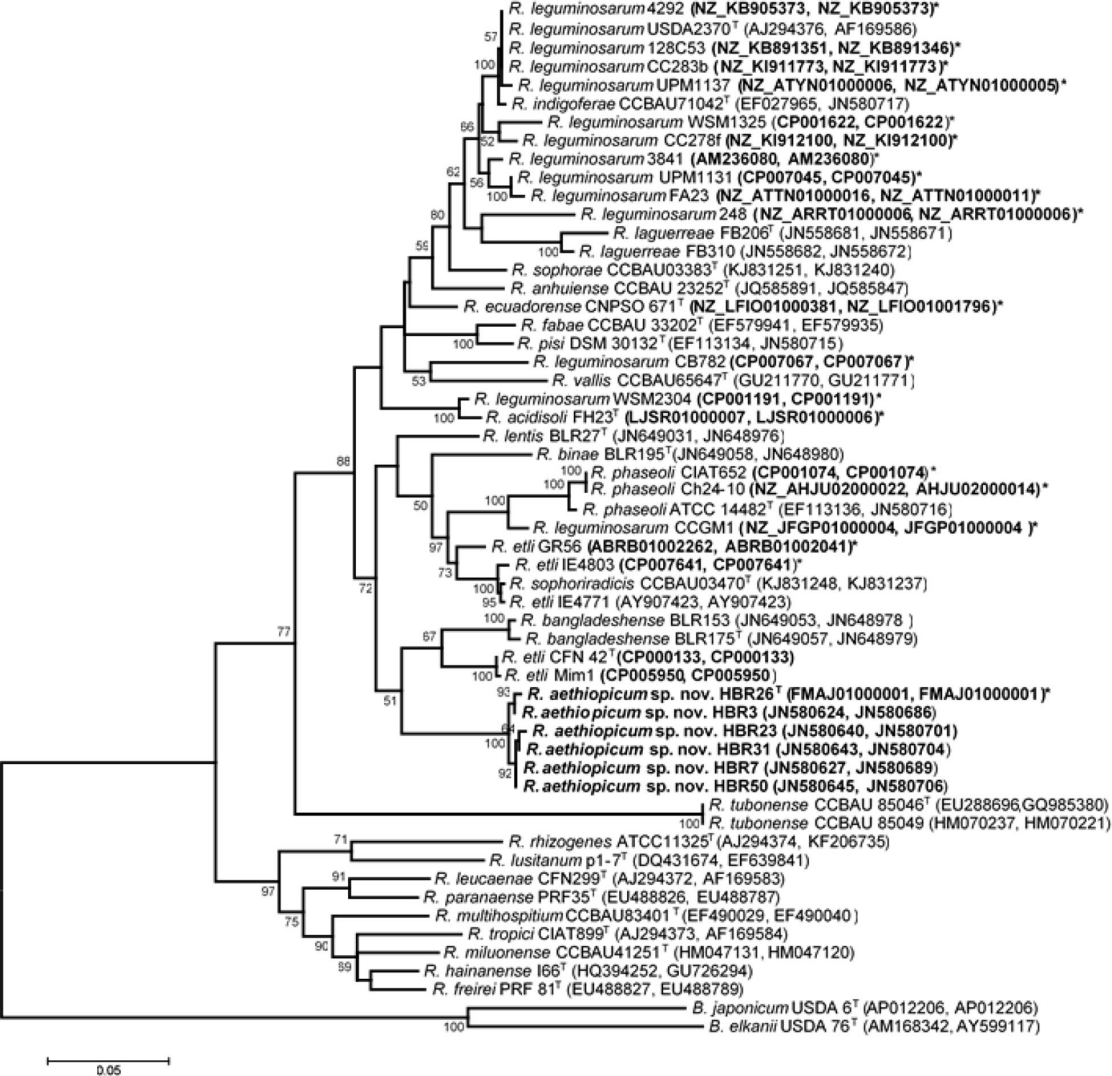
Maximum Likelihood phylogenetic tree reconstructed based on *recA-glnII* concatenated nucleotide sequences, showing the relationships between *Rhizobium aethiopicum* sp. nov. (in bold) and recognized species of the genus *Rhizobium*. The tree was constructed by using Tamura-Nei model using MEGA version 7. A discrete Gamma distribution was used to model evolutionary rate differences among sites (5 categories (+G, parameter = 0.3397). The rate variation model allowed for some sites to be evolutionarily invariable ([+I], 32.0253% sites). Bootstrap values (100 replicates) are indicated at the branching points. Reference type strains are indicated with superscript ‘T’. Bar, % estimated substitutions. GenBank accession numbers of the sequences (*recA, glnII* in order) are listed in parentheses next to strains codes. The accession numbers of whole genome sequenced strains are indicated with bold*. Abbreviations: B, *Bradyrhizobium*; R, *Rhizobium*; sp., species

The strain HBR26 ^T^ is the type strain of *R. aethiopicum* sp. nov. This strain and other strains in the novel species were isolated from nodules of common bean plants in Ethiopia. Based on multiple housekeeping gene analysis, the closest validly published species was *R. etli* [24]. In this study, a partial 16S rRNA gene tree was constructed by retrieving more and recently published reference sequences from the GenBank database. In the phylogenetic tree, the novel species grouped together and showed high 16S rRNA gene sequence similarity (99%) with strains in the neighbor groups *R. etli* CFN42 ^T^, *Rhizobium vallis* CCBAU65647 ^T^, *Rhizobium phaseoli* CIAT652, *Rhizobium pisi* DSM30132 ^T^, *Rhizobium binae* BlR195^T^, and *R. bangladeshense* BLR175^T^ (Fig. 1). We also analyzed the housekeeping genes *recA* and *glnII* to resolve the relationships between strains in novel species and known species in the *R. leguminosarum* complex group [24]. In the phylogenetic tree reconstructed based on the concatenated sequences, the novel species formed a clearly distinct group branching from the rhizobial species *R. etli* and *R. bangladeshense* (Fig. 2). This result was in agreement with our previous tree produced from concatenated partial 16S rRNA, *recA*, *rpoB* and *glnII* gene sequences [24]. Strain HBR26 ^T^ and other strains in the novel species showed high *recA* and *glnII* gene sequence (892 bp) similarities among each other. The similarities between HBR26 ^T^ and the type strains *R. etli* CFN42 ^T^ and *R. bangladeshense* BLR175^T^ ranged from 93 to 94%, CFN42 ^T^ being the closest type strain with a sequence similarity of 94%.

**Fig. 3:**
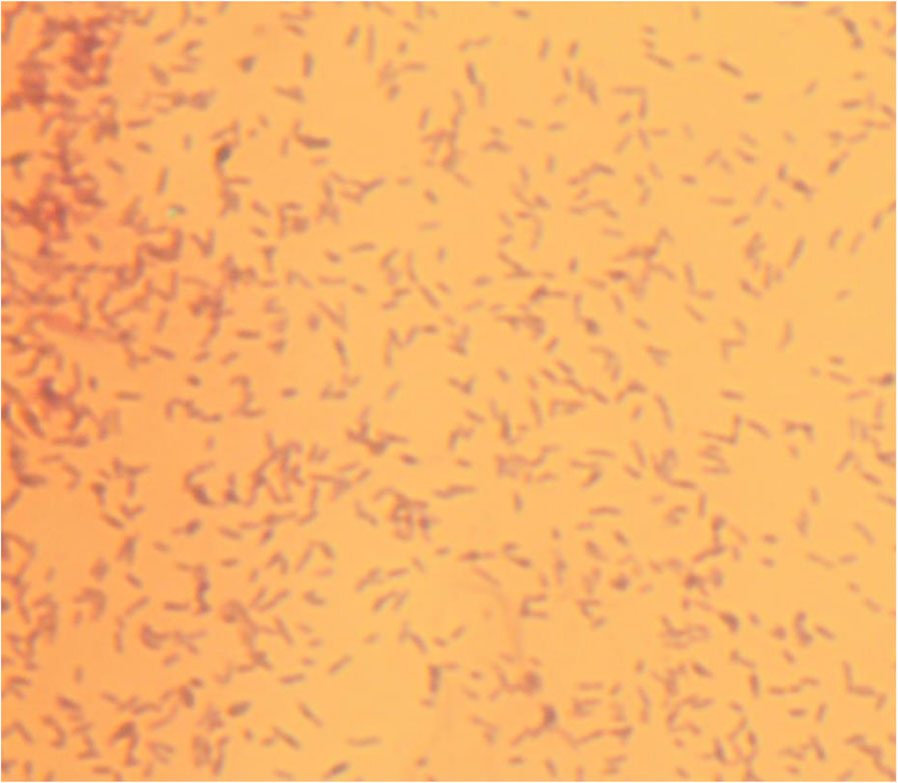
Gram stain of *Rhizobium aethiopicum* sp. nov. strain HBR26^T^

Minimum Information about the Genome Sequence is provided in Table 1 and the Additional file 1: Table S1. *R. aethiopicum* sp. nov. HBR26 ^T^ is fast-growing, forming moist, raised and smooth colonies 3–5 mm in diameter within 3–4 days on YEM agar plates at 28 °C. It is able to grow in the 15 °C to 30 °C temperature range, but its optimal growth was at 28 °C. The organism is able to grow at NaCl concentrations of 0–0.5% and at pH values in the range 5–10. Growth at pH4, at 4 °C and at 37 °C, and in 1-5% NaCl was recorded negative (Additional file 1: Table S1). This bacterial species is Gram-negative and rod shaped with a size of 1.0-2.4 μM in length (Fig. 3). HBR26 ^T^ and other strains in the novel species were able to respire many carbon sources when assessed by Biolog GN2 plates following the manufacturer’s instructions [38]. In brief, colonies grown on YEM agar were transferred to and incubated for 48–96 h at 28 °C on freshly prepared R2A media consisting of yeast extract 0.5 g, proteose peptone 0.5 g, casamino acids 0.5 g, glucose 0.5 g, soluble starch 0.5 g, sodium pyruvate 0.3 g, K_2_HPO_4_ 0.3 g, MgSO_4_.7H_2_O 0.05 g, and noble agar 15 g per liter of distilled H_2_O at pH7.2. Then colonies were suspended in 0.5% (w/v) saline (turbidity level of 52% transmittance), and 150 μl of the saline suspension was transferred to each of 96 wells of the Biolog GN2 Microplate. The plates were incubated at 28 °C, and results were checked after 4, 24, and 48 h. Positive results were recorded when the wells turned purple. All tested *R. aethiopicum* sp. nov. strains could respire 40 of the substrates in common, but 21 carbon sources were not respired by any of the tested strains. While the test strains did not show much diversity among themselves in substrate utilization pattern, they were distinctly different from carbon source respiration pattern of the closest reference *R. etli* CFN42 ^T^; the test strains responded positively for seven carbon sources that were not used by *R. etli* CFN42 ^T^. Substrates D-galactonic acid, lactone, sebacic acid and D- and L-α-glycerol phosphate were used exclusively by HBR26 ^T^. Quinic acid and glycyl-L-aspartic acid were used solely by *R. aethiopicum* sp. nov. HBR31. The details of carbon source assimilation results are presented in Additional file 2: Table S2.

**Table 1:** Classification and general features of *Rhizobium aethiopicum* sp. nov. HBR26^T^ [63]

MIGS ID	Property	Term	Evidence code
		Domain *Bacteria*	TAS [64]
		Phylum *Proteobacteria*	TAS [65]
		Class *Alphaproteobacteria*	TAS [66]
	Classification	Order *Rhizobiales*	TAS [67]
		Family *Rhizobiaceae*	TAS [68]
		Genus *Rhizobium*	TAS [68, 69]
		Species *R. aethiopicum* sp. nov.	IDA
		Type strain HBR26^T^	IDA
	Gram stain	Negative	IDA
	Cell shape	Rod	IDA
	Motility	Motile	IDA
	Sporulation	Non-sporulating	IDA
	Temperature range	Mesophile	IDA
	Optimum temperature	28 °C	IDA
	pH range; Optimum	5–10; 7	IDA
	Carbon source	Varied (see Additional file 2: Table S2)	IDA
MIGS-6	Habitat	Soil, root nodule, on host	TAS [24]
MIGS-6.3	Salinity	Non-halophile	IDA
MIGS-22	Oxygen requirement	Aerobic	IDA
MIGS-15	Biotic relationship	Free living, symbiotic	IDA
MIGS-14	Pathogenicity	Non-pathogenic	NAS
MIGS-4	Geographic location	Central Ethiopia	TAS [24]
MIGS-5	Sample collection	September, 2007	TAS [24]
MIGS-4.1	Latitude	8° 35′ 49.80″	TAS [24]
MIGS-4.2	Longitude	39° 22′ 49.27″	TAS [24]
MIGS-4.4	Altitude	1661	TAS [24]

Evidence codes – *IDA* Inferred from Direct Assay, *TAS* Traceable Author Statement (i.e., a direct report exists in the literature), *NAS* Non-traceable Author Statement (i.e., not directly observed for the living, isolated sample, but based on a generally accepted property for the species, or anecdotal evidence). These evidence codes are from the Gene Ontology project [70]

#### *Symbiotaxonomy*

HBR26 ^T^ including other strains in the *R. aethiopicum* sp. nov. are nodule forming and nitrogen-fixing on common bean host plants. The strains were originally isolated from root nodules of common bean plants growing in soils of Ethiopia [24]. In this study, the nodulation and nitrogen fixation capability was tested on legumes plants common bean, faba bean (*Vicia faba*) (http://plants.usda.gov/core/profile?symbol=VIFA), field pea (*Pisum sativum*) (http://plants.usda.gov/core/profile?symbol=PISA6) and lentil (*Lens culinaris*) (http://plants.usda.gov/core/profile?symbol=LECU2) on a sand, vermiculite and gravel mixture plant medium (5:3:3 ratio, respectively) in a growth chamber as previously described [24]. The test revealed that the strains were able to form effective nitrogen-fixing nodules in symbioses with common bean host plants. Nevertheless, the strains were not able to form symbiotic associations with faba bean, field pea and lentil. The nodulation and symbiotic characteristics results are summarized in Additional file 1: Table S1.

## Genome sequencing information

### Genome project history

In our previous study [24], the organism showed a unique phylogenetic position which most likely represented a new species. Thus, it was chosen for genome sequencing in order to describe a new species by comparing its genome sequence with the genome sequences of other close *Rhizobium* species. This project was a part of the DOE JGI 2014 Genomic Encyclopedia of Type Strains, Phase III the genomes of soil and plant-associated and newly described type strains sequencing program. The genome project is deposited at the DOE JGI genome portal [39] and also available at European Nucleotide Archive [40] under accession numbers FMAJ01000001-FMAJ01000062. Sequencing, assembling, and annotation were done by the DOE JGI. A summary of the genome project information is listed in Table 2.

**Table 2:** Project information

MIGS ID	Property	Term
MIGS 31	Finishing quality	High-quality draft
MIGS-28	Libraries used	Illumina std shotgun library
MIGS 29	Sequencing platforms	Illumina HiSeq 2500, Illumina HiSeq 2500-1 TB
MIGS 31.2	Fold coverage	258.1×
MIGS 30	Assemblers	Velvet (version 1.2.07), Allpaths–LG (version r46652)
MIGS 32	Gene calling method	Prodigal
	Locus Tag	ATF61
	Genbank ID	FMAJ00000000
	Genbank Date of Release	03-AUG-2016
	GOLD ID	Gp0108286
	BIOPROJECT	PRJNA303274
MIGS 13	Source Material Identifier	HBR26
	Project relevance	Symbiotic N_2_ fixation, agriculture

### Growth conditions and genomic DNA preparation

First HBR26 ^T^ (=HAMBI 3550 ^T^=LMG 29711 ^T^) was grown aerobically on YEM agar plates at 28 °C. A pure colony was transferred into 3 ml YEM broth medium and the cell culture was grown for four days in a shaker incubator (200 rpm) at 28 °C. One ml was used to inoculate 150 ml YEM broth, and cells were grown on a shaker (200 rpm) again at 28 °C until the culture reached late-logarithmic phase. DNA was isolated from cell pellets collected in a 60 ml following the CTAB bacterial genomic DNA isolation protocol Version Number 3 provided by the DOE JGI [41].

### Genome sequencing and assembly

The genome was sequenced at the DOE JGI using a combination of Illumina HiSeq 2500 and Illumina HiSeq 2500-1 TB technologies [42]. An Illumina standard shotgun library was constructed and sequenced using the Illumina HiSeq 2000 platform which generated 9,310,748 reads totaling 1405.9 Mbp. Methods used for library construction and sequencing can be found at the DOE JGI website [43]. In order to discard artifacts from Illumina sequencing and library preparation, all raw Illumina sequence data was passed through the program DUK at DOE JGI [43]. Filtered Illumina reads were assembled using Velvet (version 1.2.07) [44] and then from Velvet contigs, 1–3 kb simulated paired-end reads were constructed using wgsim (version 0.3.0) (https://github.com/lh3/wgsim). Allpaths–LG (version r46652) [45] was used to assemble Illumina reads with a simulated read. The final assembly was based on 1,290.5 Mbp of Illumina data, which provides 258.1× input read coverage of the genome. The draft genome is 6.6 Mbp in size and contains 64 contigs in 62 scaffolds.

### Genome annotation

Genes were predicted using Prodigal [46] and using the DOE JGJ annotation pipeline [47]. The identified protein-coding genes were translated and functionally annotated by comparing the sequences with the NCBI non-redundant database, UniProt, TIGRFam, Pfam, KEGG, COG, and InterPro databases. The tRNA genes were found using tRNAScanSE tool [48] and ribosomal RNA genes were identified by searches against models of the ribosomal RNA genes at the SILVA database [49]. Other non–coding RNAs such as the RNA components of the protein secretion complex and the RNase P were identified by searching the genome for the corresponding Rfam profiles using INFERNAL [50]. Additional analysis was accomplished using the IMG tool [51]. The same tool was also used for manual functional annotation of the predicted genes and for examining the genome sequence.

## Genome properties

The genome of HBR26 ^T^ is arranged in 62 scaffolds and consists of 6,557,588 bp, with a 61% G + C content. In total 6307 genes were predicted, of these 6221 were protein-coding genes and 86 were RNA genes. Five rRNAs identified including one 16S rRNA, two 5S rRNA, and two 23S rRNA genes. There were 52 tRNA genes and 29 other (miscRNA) RNA genes. The statistics and properties of the genome are summarized in Table 3. The majority of the protein-coding genes, 5054 (80.13%) were assigned with putative functions (Table 3), and of these 4578 genes (72.59%) were assigned to COG functional categories (Table 4). The remaining genes were annotated as hypothetical proteins (1167 genes, 18.5%).

**Table 3:** Genome statistics

Attribute	Value	% of total
Genome size (bp)	6,557,588	100
DNA coding (bp)	5,707,275	87.03
DNA G + C (bp)	4,004,707	61.07
DNA scaffolds	62	100
Total genes	6307	100
Protein coding genes	6221	98.64
RNA genes	86	1.36
Pseudo genes	not determined	
Genes in internal clusters	962	15.25%
Genes with function prediction	5054	80.13%
Genes assigned to COGs	4578	72.59%
Genes with Pfam domains	5315	84.27%
Genes with signal peptides	530	8.40%
Genes with transmembrane helices	1406	22.29%
CRISPR repeats	0

**Table 4:** Number of genes associated with general COG functional categories

Code	Value	%age	Description
J	221	4.24	Translation, ribosomal structure and biogenesis
A	0	00	RNA processing and modification
K	467	8.96	Transcription
L	123	2.36	Replication, recombination and repair
B	2	0.04	Chromatin structure and dynamics
D	41	0.79	Cell cycle control, Cell division, chromosome partitioning
V	115	2.21	Defense mechanisms
T	252	4.83	Signal transduction mechanisms
M	274	5.25	Cell wall/membrane biogenesis
N	85	1.63	Cell motility
U	106	2.03	Intracellular trafficking and secretion
O	189	3.62	Posttranslational modification, protein turnover, chaperones
C	267	5.12	Energy production and conversion
G	557	10.68	Carbohydrate transport and metabolism
E	557	10.68	Amino acid transport and metabolism
F	108	2.07	Nucleotide transport and metabolism
H	239	4.58	Coenzyme transport and metabolism
I	209	4.01	Lipid transport and metabolism
P	274	5.25	Inorganic ion transport and metabolism
Q	145	2.78	Secondary metabolites biosynthesis, transport and catabolism
R	566	10.83	General function prediction only
S	363	6.96	Function unknown
-	1729	27.41	Not in COGs

The total is based on the total number of protein coding genes in the genome

## Insights from the genome sequence

### Genome wide comparative analysis

Based on *recA-glnII* concatenated sequence comparisons, the proposed type strain HBR26 ^T^ and strains included in *R. aethiopicum* sp. nov., HBR23, HBR3, HBR31, HBR7, and HBR50 were closely related to each other (99–100% sequence identity). Nevertheless, these strains were only distantly related to the closest reference strains *R. etli* CFN42 ^T^ (94%) and *R. bangladeshense* BLR175^T^ (93%). In order to further resolve the taxonomy of the novel group, genomic comparative analyses were done between HBR26 ^T^ and several relatively close reference strains presented in the Fig. 2. For this the genomes of a number strains, such as *R. etli* CFN42 ^T^, *Rhizobium etli* IE4771, *Rhizobium etli* Mim1, *Rhizobium etli* IE4803, *Rhizobium phaseoli* Ch24-10, *Rhizobium phaseoli* CIAT652, *Rhizobium acidisoli* FH23, *Rhizobium ecuadorense* PSO671 ^T^, and *Rhizobium leguminosarum* CB782, CCGM1, WSM2304, PM1131, WSM1325, 4292, 3841, and UPM1137 were retrieved from the DOE JGI genome portal (Tables 5 and 6). ANI was computed from protein-coding genes of the genomes using the MiSI program implemented in the IMG database [35]. For a pair of genome sequences, the system calculates ANI by averaging the nucleotide identity of orthologous genes identified as bidirectional best hits and also calculates Alignment Fraction of orthologous genes [35]. In addition, partially sequenced genome reads from *R. bangladeshense* BLR175^T^, *Rhizobium lentis* BLR27^T^, *Rhizobium binae* BLR 95^T^, *Rhizobium anhuiense* CCBAU23252 ^T^, *R. pisi* DSM30132 ^T^ and *Rhizobium fabae* CCBAU33202 ^T^ were used for calculation of additional ANI with the JSpecies program using default parameters as previously used [52, 53]. Table 5 shows the ANI values obtained between HBR26 ^T^ and reference strains (numbers above the diagonal). The numbers below the diagonal show pairwise orthologous genes identified as bidirectional best hits between genomes. AF was >0.68 in all ANI calculations among whole or draft genomes but the AF value was <0.6 in all ANI calculations with partially sequenced genome reads. The ANI values obtained between HBR26 ^T^ and references strains varied between 87.4 and 91.8%, which was below 96%, the value of relatedness recommended for species delineation [35]. The closest strains were *R. bangladeshense* LR175^T^ and *R. etli* CFN42 ^T^ with ANI values 91.8 and 90.2%, respectively. This result is in agreement with the *recA-glnII* concatenated analysis (Fig. 2), confirming that that HBR26 ^T^ is distantly related to the *R. etli* and *R. bangladeshense* species but belongs to the novel *Rhizobium* species. The ANI between *R. etli* IE4803 and *R. etli* IE4771 was 97.7%. However, ANI values between these strains and the type strain *R. etli* CFN42 ^T^ (= < 90.2%) was much below the cutoff value of strains of the same species. Several *R. leguminosarum* strains included in Table 5 may represent species other than *R. leguminosarum* (ANI < 96% each other). The genome of *R. leguminosarum* CCGM1 showed a significantly higher degree of similarity with *R. phaseoli* Ch24-10 (97.2% ANI) and CIAT652 (97.2% ANI), and could thus be classified as *R. phaseoli*. *R. leguminosarum* WSM2304 showed 96.6% genomic relatedness with *R. acidisoli* FH23^T^. Accordingly, we suggest the classification of WSM2304 under *R. acidisoli* species. The ANI value between *R. fabae* CCBAU33202 ^T^ and *R. pisi* DSMZ30132 ^T^ was 96.6%. This value corroborates the relationship between the two strains as reported previously [24], which is also shown in the *recA-glnII* based phylogenetic tree in Fig. 2, suggesting that *R. fabae* CCBAU33202 ^T^ and *R. pisi* DSMZ30132 ^T^ might represent one and the same species.

**Table 5:**
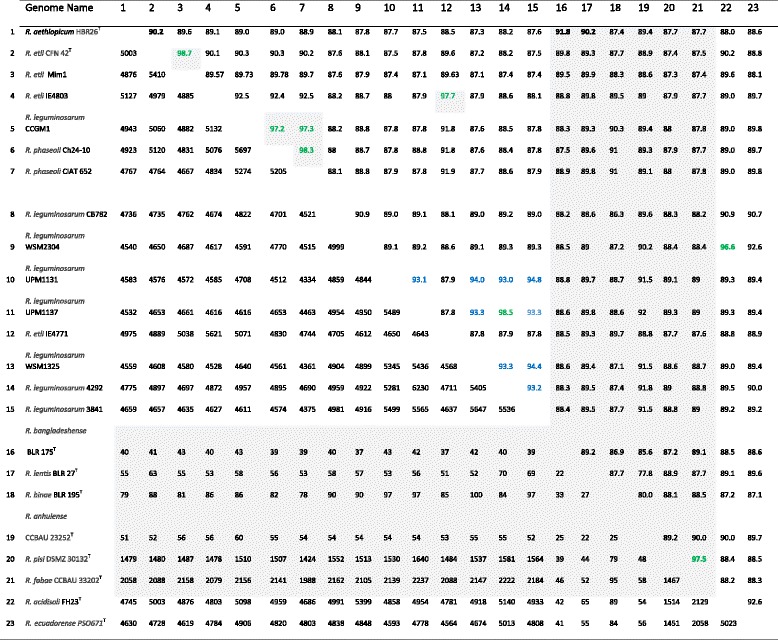
ANI Genomic comparison between *R. aethiopicum* sp. nov. HBR26^T^ and other members of *Rhizobium* species

Gray shade indicates ANI calculated using partially sequenced genomes. The conting fatsa files of the reads were obtained from Professor J.P.W. Young, the University of York and read data are also deposited at NCBI database under Bioproject accession number PRJEB7125 or PRJEB7987; number below the diagonal are pairwise orthologous genes identified as bidirectional best hits between genomes; AF was >0.68 in all ANI calculation among whole or draft genome but AF value was <0.6 in all ANI calculation with partially sequenced genome reads. Numbers above the diagonal are ANI between genomes. Reference type strains are indicated with superscript ‘T’; R, *Rhizobium*

**Table 6:** Genome statistics of *R. aethiopicum* sp. nov. HBR26^T^ and reference rhizobial strains

Status	Genome Name	IMG Genome ID	GenBank Accession number	Quality	Host Name	Genome Size (Mbp)	Gene	Scaf- fold^a^	GC %	CDS %	RNA^a^	COG %	KOG %	Pfam %	TIGR- fam %	KEGG %
Draft	*R. aethiopicum* BR26^T^	2615840624	PRJNA303274	High	*P. vulgaris* (http://plants.usda.gov/core/profile?symbol=PHVU)	6.6	6307	62	0.61	98.64	86	72.6	18.1	84.3	24.8	29.7
Finished	*R. etli* CFN 42^T^	2623620267	CP000133	High	*P. vulgaris* (http://plants.usda.gov/core/profile?symbol=PHVU)	6.5	6345	7	0.61	98.5	95	69.9	17.5	82.0	24.2	29.0
Finished	*R. etli* Mim1	2565956559	CP005950	High	*Mimosa affinis* ^*b*^	7.2	7006	7	0.61	97.82	153	70.2	17.8	80.2	24.3	28.7
Finished	*R. etli* IE4803	2630968325	CP007641	High	*P. vulgaris* (http://plants.usda.gov/core/profile?symbol=PHVU)	7.0	6708	5	0.61	98.57	96	71.3	17.6	83.0	24.6	29.0
P. Draft	*R. leguminosarum* CCGM1	2609460209	JFGP00000000	High	*P. vulgaris* (http://plants.usda.gov/core/profile?symbol=PHVU)	6.9	6711	55	0.61	98.63	92	69.2	17.1	81.0	23.8	28.1
P. Draft	*R. phaseoli* Ch24-10	2548876814	AHJU00000000	High	*P. vulgaris* (http://plants.usda.gov/core/profile?symbol=PHVU)	6.6	6593	352	0.61	98.82	78	67.6	16.6	81.0	23.7	28.2
Finished	*R. phaseoli* CIAT 652	642555152	CP001074	High	*P. vulgaris* (http://plants.usda.gov/core/profile?symbol=PHVU)	6.5	6132	4	0.61	99.02	60	70.7	17.7	81.2	25.2	29.0
Finished	*R. leguminosarum* CB782	2510065076	CP007067	High	*Trifolium semipilosum* (http://plants.usda.gov/core/profile?symbol=TRSE7)	6.7	6559	4	0.61	98.67	87	72.5	18.4	83.2	24.5	28.8
Finished	*R. leguminosarum* WSM2304	643348569	CP001191	High	*T. polymorphum* (http://plants.usda.gov/java/ClassificationServlet?source=display&classid=TRPO6)	6.9	6643	5	0.61	99.07	62	70.9	18.6	83.1	23.9	28.5
P. Draft	*R. leguminosarum* UPM1131	2513237084	CP007045	High	*Pisum* *sativum* (http://plants.usda.gov/core/profile?symbol=PISA6)	7.2	6951	41	0.61	98.83	81	72.9	17.9	83.5	23.7	27.9
P. Draft	*R. leguminosarum* UPM1137	2513237085	ATYN00000000	High	*P. sativum* (http://plants.usda.gov/core/profile?symbol=PISA6)	7.7	7462	49	0.61	99.04	72	71.0	17.6	81.9	22.4	28.1
Finished	*R. etli* IE4771	2585427632	CP006986	High	*P. vulgaris* (http://plants.usda.gov/core/profile?symbol=PHVU)	7.1	6894	6	0.61	98.23	122	71.3	17.9	81.1	24.3	28.9
Finished	*R. leguminosarum* WSM1325	644736401	CP001622	High	*Trifolium* (http://www.theplantlist.org/tpl1.1/record/ild-8146)	7.4	7292	6	0.61	99.18	60	68.7	17.5	81.6	22.4	26.9
P. Draft	*R. leguminosarum* 4292	2516653085	AQZR01000000	High	*P. vulgaris* (http://plants.usda.gov/core/profile?symbol=PHVU)	7.3	7193	5	0.61	98.83	84	71.8	17.9	83.2	23.2	28.5
Finished	*R. leguminosarum* 3841	2623620212	AM236080	High	*P. sativum* (http://plants.usda.gov/core/profile?symbol=PISA6)	7.8	7447	7	0.61	98.74	94	71.7	17.7	82.7	22.5	27.3
P. Draft	*R.acidisoli* FH23^T^	2648501703	LJSR00000000	High	*P. vulgaris* (http://plants.usda.gov/core/profile?symbol=PHVU)	7.3	7111	104	0.61	98.83	83	69.6	17.4	81.6	22.9	27.6
P. Draft	*R. ecuadorense* CNPSO 671^T^	2648501138	LFIO00000000	High	*P. vulgaris* (http://plants.usda.gov/core/profile?symbol=PHVU)	6.9	6668	139	0.61	98.85	77	71.2	17.8	82.3	24.2	29.1

P. draft, permanent draft; ^*a*^ number of scaffolds or number of RNA; ^b^broad host range, including plants of *M. affinis* (http://www.theplantlist.org/tpl1.1/record/ild-15931), *Leucaena leucocephala* (http://plants.usda.gov/core/profile?symbol=LELEL2), *Calliandra grandiflora* (http://www.theplantlist.org/tpl1.1/record/ild-20119), *Acaciella angustissima* (http://www.theplantlist.org/tpl1.1/record/ild-28474) as well as *P. vulgaris* [71]. Reference type strains are indicated with superscript ‘T’; R, *Rhizobium*

Table 6 shows the genome statistics and functional category comparison between HBR26 ^T^ and close reference rhizobial strains. The draft genome of HBR26 ^T^ (6.6 Mbp) is about the same size as that of *R. phaseoli* Ch24-10 (6.6 Mbp) and slightly greater than *R. etli* CFN42 ^T^ (6.5 Mbp) and *R. phaseoli* CIAT652 (6.4 Mbp). However, strain HBR26 ^T^ has smaller genome size compared to *R. leguminosarum* CCGM1 (6.8 Mbp), *R. etli* IE4803 (6.9 Mbp), *R. acidisoli* FH23 (7.3Mbp), *R. ecuadorense* CNPSO671 (6.9Mbp) and all other *R. leguminosarum* (6.8-7.9 Mbp) symbiovar *viciae* and *trifolii* reference strains (Table 6). Though the gene content of strain HBR26 ^T^ (6307) is only greater than of CIAT652 (6132), it has got the highest percentage of genes assigned to Pfam (84.3%), TIGRfam (24.8%), and KEGG (29.7%). HBR26 ^T^ also has the highest percentage of genes assigned to COG (72.6%) and KOG (18.1%) functional categories, with the exceptions *R. leguminosarum* UPM1131 (72.9%), and WSM2304 (18.6%), respectively.

**Fig. 4:**
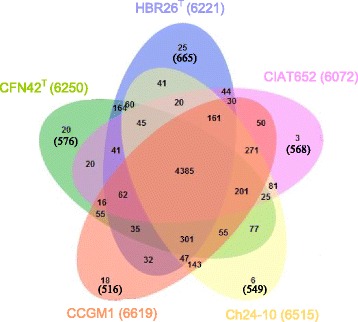
Venn diagram plotted by OrthoVenn program shows shared orthologous protein clusters among the genomes of bean-nodulating rhizobial strains (in the center): *Rhizobium etli* CFN42^T^, *Rhizobium phaseoli* Ch24-10, *Rhizobium phaseoli* CIAT652, *Rhizobium leguminosarum* CCGM1 and *Rhizobium aethiopicum* type strain HBR26^T^. The number of protein clusters comprising multiple protein families is indicated for each genome and also the number of singletons i.e., protein with no orthologous of each strain are shown in parenthesis. The total number of protein sequences of each genome are indicated in parentheses next to strains codes

In Fig. 4 the Venn diagram plotted in the OrthoVenn program shows overlapping orthologous protein clusters between the genomes of HBR26 ^T^ and other common bean-nodulating references *R. etli* CFN42 ^T^, and *R. phaseoli* Ch24-10, CIAT652 and CCGM1. The orthologous clusters were identified with default parameters, 1e-5 e-value cutoff for all protein similarity comparisons and 1.5 inflation value for the generation of orthologous clusters [54]. In total the strains formed 6534 protein clusters, 6462 orthologous clusters (at least containing two strains) and 4273 single-copy gene clusters. All five strains shared in common 4385 orthologous protein clusters. On a pairwise basis, HB26^T^ shares 32, 42 and 44 proteins with CCGM1, Ch24-10, and CIAT652, respectively. Strain HBR26 ^T^ shares the most with CFN42 ^T^ with 164 orthologous group. This result is in agreement with *recA-glnII* phylogenetic and ANI analysis, supporting that HBR26 ^T^ is more closely related to CFN42 ^T^ compared to the other bean-nodulating strains. The genome of HBR26 ^T^ contains the highest number of genome-specific proteins of the five strains with 665 singletons followed by CFN 42 ^T^, CIAT652, Ch24-10 and CCGM1 with 568, 549 and 516 singletons, respectively.

### Comparative analysis of accessary genes: emphasis on symbiotic genes

Genes which are not essentially present in all bacterial strains are known as accessory genes. These genes are contained by mobile elements such as plasmids, genomic islands, transposons or phages and thus can be gained or lost among bacterial strains through horizontal gene transfer mechanisms. Accessory genes in the genome of HBR26 ^T^ were searched by assembling against the reference genome *R. etli* CFN42 ^T^ using the Genome Gene Best Homologs package from program IMG-ER [55]. Additional file 3: Table S3 shows homologous *repABC* (plasmid replication genes) and symbiotic genes found in the genome of HBR26 ^T^. The result revealed that HBR26 ^T^ carries five different *repABC* genes homologous to the genes found in five of the *R. etli* CFN42 ^T^ [56] plasmids 42b, 42c, 42d, 42e, 42f, suggesting that HBR26 ^T^ may have five additional replicons other than the chromosome. The *repABC* genes corresponding to the symbiotic plasmid 42d showed high sequence similarity between other common bean nodulating strains CFN42 ^T^, CIAT652, IE4803, Ch24-10, 4292 and CCGM1 (identity ranging 99–100%). This implies that bean- nodulating strains and HBR26 ^T^ may share common symbiotic plasmids. The HBR26 ^T^ *repABC* genes homologous to 42b, 42c, 42e and 42f also showed sequence similarity in the ranges 86–89%, 84–93%, 92–94%, 84–93%, respectively, with strains CIAT652, CFN42 ^T^, IE4803, Ch24-10, 4292, CCGM1 and *R. etli* sv. *mimosae* Mim1.

The symbiosis between rhizobia and legume plants is initiated when plant exudates known as flavonoids trigger expression of the rhizobial nodulation genes that code for the synthesis of LCO Nod factors. The backbone of this LCO is encoded by the common *nodABC* accessory genes. There are also additional genes (*nol*, *noe*) which code for the substituent groups that decorate the LCO core [57]. The symbiosis between rhizobia and legumes results in the formation of specialized organs on plant roots known as nodules in which rhizobia differentiate into N_2_-fixing bacteroids [58]. Like most symbiotic rhizobia, the genome of HBR26 ^T^ carries the symbiotic genes encoding for the synthesis of LCO structures, substituent groups and genes coding for nitrogen fixation (Additional file 3: Table S3). Several of the nodulation and nitrogen-fixing genes are located on the scaffolds Ga0061105_135 and Ga0061105_130, 141, 144 and 150. The first scaffold contains the main nodulation genes except *nodA,* while the other scaffolds encompass many of the nitrogen-fixing genes (Additional file 3: Table S3).

**Fig. 5:**
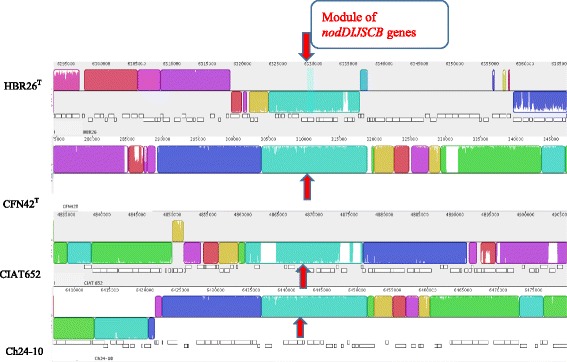
Mauve alignment comparing the genome of *Rhizobium aethiopicum* type strain HBR26^T^ with the genome of *Rhizobium etli* CFN42^T^, *Rhizobium phaseoli* CIAT652 and *Rhizobium phaseoli* Ch24-10. The module of *nodDIJSCB* genes are indicated by the *arrows*

The genomes of HBR26 ^T^, *R. etli* CFN42 ^T^, *R. phaseoli* Ch24-10 and CIAT652 were aligned using the progressive Mauve alignment tool [59], using default parameters. The genomic features were visualized using the Artemis Comparison Tool [60, 61]. The Mauve alignment in Fig. 5 shows the presence of a similar *nodBCSIJD* module organization between the genome of HBR26 ^T^ and the genomes of other bean-nodulating rhizobial strains CFN42 ^T^, CIAT652, and Ch24-10. The *nodDIJSCB* genes are flanked by transposase genes and hypothetical protein-coding genes. A similar arrangement of the *nod* genes was also found in the genomes of CCGM1 and IE4803, which are also micro-symbionts of common bean (data not shown).

All HBR26 ^T^, CFN42 ^T^, Ch24-10, CIAT652, and CCGM1 genomes carry additional *nodZ, noeI* and *nolE* genes adjacent to the *nodBCSIJD* region. Similarly, in the genomes of clover and faba bean nodulating *R. leguminosarum* WSM2304, UPM1131 and 3841 the nodulation genes *nodD, nodB, nodC, nodI,* and *nodJ* are also clustered in the same region. In the latter case, this region contains additional *nodA, nodL, nodE,* and *nodF* genes as well. The *nodA* and *nolL* genes of HBR26 ^T^ which are located in the scaffolds Ga0061105_134 and Ga0061105_130, respectively, are very similar to the corresponding gene sequences of bean-nodulating rhizobial strains CFN42 ^T^, Ch24-10, CCGM1, CIAT652 and IE4803 (99–100% similarity). Its *nodB* gene is also homologous with CFN42 ^T^, CIAT652, and IE4803. The highest identity (100%) is with *nodB* of IE4803 followed by CFN42 ^T^ (98%) and CIAT652 (97%). *nodC* of HBR26 ^T^ shares 97% similarity with *nodC* of CIAT652, CFN42 ^T^ and, CCGM1. All *nodS, nodI* and *nodJ* genes of HBR26 ^T^ share high identity with those of CIAT652 (99%), CFN42 ^T^ (98%), CCGM1 (98%) and Ch24-10 (98%).

The nitrogenase complex, an enzyme responsible for nitrogen fixation in diazotrophs, consists of two components known as dinitrogenase and dinitrogenase reductase [62]. The *nif* genes are required for the synthesis and functioning of the nitrogenase complex [62]. Many of these genes in the genome of HBR26 ^T^ are harbored in four different scaffolds Ga0061105_130, Ga0061105_150, Ga0061105_144, and Ga0061105_141. The first scaffold contains the *nifA-nifB-nifT-nifZ-nifW* genes, and the second scaffold includes the *nifE, nifN* and *nifX* genes. The nitrogen-fixing genes *nifH, nifU* and *nifQ* are retained in the scaffold Ga0061105_141. An additional *nifH* gene, *fixG* and *fixH* genes are found in the scaffold Ga0061105_144 and a *nifK* gene is located in the scaffold Ga0061105_162. The dinitrogenase component of the nitrogenase complex is a product of *nifD* and *nifK* genes and the dinitrogenase reductase is coded by *nifH* [62]. However, the *nifD* gene is missing in the draft genome of HBR26 ^T^. This gene is important to enable the nitrogenase enzyme complex functional. On the other hand, the strain HBR26 ^T^ makes effective nitrogen-fixing symbiosis with common bean plants. Thus, the reason behind the absence of *nifD* in the genome of HBR26 ^T^ is probably because our data is a draft genome and probably *nifD* was missed during sequencing. It is also possible that *nifD* sequence was truncated when the library was constructed.

The genes *nifB, nifT, nifZ, nifE, nifN, nifX, fixG, fixH, nifW, nifQ, nifK* and *nifH* all share high identity with homologous genes found in CFN42 ^T^ (98–100%), Ch24-10 (98–100%), CCGM1 (98–100%), 4292 (96–99%) or in IE4803 (92–100%). In our previous study, we identified rhizobial strains belong to *R. phaseoli*, *R. etli* and *R. leguminosarum* from root nodules of common bean plants growing in the soils of Ethiopia [24]. Thus, the close similarity of the *nod*, *nif* and *fix* genes between HBR26 ^T^ and bean-nodulating *R. etli* *,* *R. phaseoli* and *R. leguminosarum* strains suggests that those genes might be shared between these rhizobial species through horizontal gene transfer mechanisms.

## Conclusion

This study presents the genome sequence for the *R. aethiopicum* sp. nov. strain HBR26 ^T^. The result from phylogenetic analyses of multilocus sequences of core genes showed a novel species within the genus *Rhizobium*. This result was further supported by ANI calculation, in which the genome of the type strain HBR26 ^T^ exhibited < 91.8% identity when compared with the genomes of close *Rhizobium* species. This value is much lower than the 96% ANI limit for delineating a species. The data confirms that *R. aethiopicum* sp. nov. should be considered as a new *Rhizobium* species. Thus, on the basis of phylogenetic, comparative genomic analyses and ANI results and by including phenotypic characteristics, we formally propose the creation of *R. aethiopicum* sp. nov. that contains the strain HBR26 ^T^ (= HAMBI 3550 ^T^=LMG 29711 ^T^). The strains included in this species are effective nitrogen-fixing rhizobia in symbiosis with common bean plants. The genome of the type strain HBR26 ^T^ carries five plasmid replication *repABC* genes homologous to the genes found in five of the *R. etli* CFN42 ^T^ plasmids, suggesting that HBR26 ^T^ may have five additional replicons other than the chromosome. The organization of *nodBCSIJD* genes is similar between the genomes of HBR26 ^T^ and other bean-nodulating rhizobial species. The symbiotic genes necessary for nodulation and for nitrogen fixation share high sequence similarity between bean-nodulating strains, such as *R. etli* *,* *R. phaseoli* and *R. leguminosarum*, which suggests that these genes might be shared between bean-nodulating rhizobial species through horizontal gene transfer mechanisms.

## Description of *Rhizobium**aethiopicum* sp. nov.

*Rhizobium aethiopicum* (ae.thi.o’pic.um. L. neut. adj. *aethiopicum*, pertaining to Ethiopia). Fast-growing, forming moist, raised and smooth colonies 3–5 mm in diameter within 3–4 days on YEM agar plates under optimal growth conditions, at 28 °C and pH7. The strains are able to grow between 15 °C and 30 °C. The organisms require no or trace amounts of NaCl for growth and are only able to grow at NaCl concentrations of 0–0.5% and at pH values in the range 5–10. No growth occurred at pH4, at temperature 4 °C and at 37 °C, and 1–5% NaCl. Cells are Gram-negative rod-shaped and 1.0–2.4 μM in length. Oxidation of the following substrates as carbon sources in Biolog GN2 microplates was recorded positive; dextrin, glycogen, N-acetyl-D-glucosamine, adonitol, L-arabinose, D-arabitol, D-cellobiose, I-erythritol, D-fructose, L-fucose, D-galactose, α-D-glucose, α-D-lactose, lactulose, maltose, D-mannitol, D-mannose, D-melibiose, β-methyl-D-glucoside, D-psicose, D-raffinose, L-rhamnose, D-sorbitol, sucrose, D-trehalose, turanose, xylitol, pyruvic acid methyl ester, succinic acid mono-methyl-ester, β-hydroxybutyric acid, γ-hydroxybutyric acid, itaconic acid, α-keto butyric acid, α-keto glutaric acid, D,L-lactic acid, succinic acid, bromo-succinic acid, succinamic acid, L-alaninamide, D-alanine, L-alanine, L-alanyl-glycine, L-asparagine, L-aspartic acid, L-glutamic acid, glycyl-L-glutamic acid, L-histidine, hydroxy-L-proline, L-ornithine, L-proline, D,L-carnitine, γ-amino butyric acid, urocanic acid, nosine, uridine, thymidine, glycerol, α-d-glucose-1-phosphate and D-glucose-6-phosphate. However, the oxidation was negative for the following substrates: α-cyclodextrin, Tween 40, Tween 80, N-acetyl-D-galactosamine, gentiobiose, acetic acid, D-galacturonic acid, D-gluconic acid, D-glucosaminic acid, D-glucuronic acid, p-hydroxy phenylacetic acid, α-keto valeric acid, propionic acid, D-saccharic acid, glucuronamide, L-phenylalanine, L-pyroglutamic acid, D-serine, phenyethyl-amine, putrescine, and 2-aminoethanol. The type strain HBR26 ^T^ (= HAMBI 3550 ^T^ =LMG 29711 ^T^) was isolated from root nodules of common bean plants growing in Ethiopia. The genome size of the type strain is 6.6 Mbp and the G + C content of the genome is 61%. The genome sequence of the type strain is deposited at DOE JGI genome portal under IMG genome/Taxon ID: 2615840624 [39] and also available at European Nucleotide Archive [40] under accession numbers FMAJ01000001-FMAJ01000062. The type strain has been deposited in the HAMBI (HAMBI 3550 ^T^) and LMG (LMG 29711 ^T^) culture collections.

## Additional files

Additional file 1: Table S1. Phenotypic characteristics of *Rhizobium aethiopicum* sp. nov. strains. (DOCX 21 kb)
Additional file 2: Table S2. Carbon sources utilization response between *Rhizobium aethiopicum* sp. nov. strains and *Rhizobium etli* CFN42^T^. (DOCX 25 kb)
Additional file 3: Table S3. Rhizobium aethiopicum sp.nov. strain HBR26T repABC and symbiotic genes homologous to genes found in the symbiotic plasmid 42d of Rhizobium etli CFN 42T. (XLSX 45 kb)

## Abbreviations

AF: Alignment fraction
ANI: Average nucleotide identity values
CTAB: Cetyl Trimethyl Ammonium Bromide
DDH: DNA-DNA Hybridization
DOE: Department of energy
GOLD: Genomes online database
IMG: Integrated microbial genomes
IMG-ER: Integrated microbial genomes – expert review
JGI: Joint Genome Institute
LCO: Lipochito-Oligosaccharide
MIGS: Minimum information about a genome sequence
MiSI: Microbial species identifier
MLSA: Multilocus sequence analysis
N_2_: Dinitrogen
R2A: Reasoner’s 2A Agar
YEM: Yeast Extract Mannitol
