# Supplementary material for: Draft genome sequence of type strain HBR26T and description of Rhizobium aethiopicum sp. nov
Source: Stand Genomic Sci. 2017 Jan 26;12:14. doi: 10.1186/s40793-017-0220-z (PMC5278577; doi:10.1186/s40793-017-0220-z)
Supplement: Additional file 1: Table S1. — Phenotypic characteristics of Rhizobium aethiopicum sp. nov. strains. (DOCX 21 kb) [file 40793_2017_220_MOESM1_ESM.docx]

Additional file 1 Table S1. Phenotypic characteristics of *Rhizobium aethiopicum* sp. nov. strains

|  | | HBR26^T^ | HBR23 | HBR31 | HBR3 |
| --- | --- | --- | --- | --- | --- |
| Temperature (3-6 days) | |  |  |  |  |
| 5^O^C | | - | - | - | - |
| 15^O^C | | **+** | **+** | **+** | **+** |
| 20 ^O^C | | **+** | **+** | **+** | **+** |
| 28-30 ^O^C | | **+** | **+** | **+** | **+** |
| 37 ^O^C | | - | - | - | - |
| 45 ^O^C | | - | - | - | - |
| pH4 (day3-7) | | - | - | - | - |
| pH5 (day3) | | **+** | **+** | **+** | **+** |
| pH9 (day3) | | **+** | **+** | **+** | **+** |
| pH10 | |  |  |  |  |
| day3 | | - | - | - | - |
| day4 | | - | - | - | - |
| day5 | | **+** | **+** | **+** | **+** |
| **NaCl** | |  |  |  |  |
| 0% (day3) | | **+** | **+** | **+** | **+** |
| 0.5% (day3) | | **+** | **+** | **+** | **+** |
| 1% (day3-7) | | - | - | - | - |
| 2% (day3-7) | | - | - | - | - |
| 3% (3-7days) | | - | - | - | - |
| 5% (3-5days) | | - | - | - | - |
| **Nodulation Test** | | | | | |
| Legumes |  |  |  |  |  |
| **Common bean** | nod | **+** | **+** | **+** | **+** |
|  | fix | **+** | **+** | **+** | **+** |
| **Faba bean** | nod | - | - | - | - |
|  | fix | - | - | - | - |
| **Lentil** | nod | - | - | - | - |
|  | fix | - | - | - | - |
| **Field pea** | nod | - | - | - | - |
|  | fix | - | - | - | - |

+, growth; nod, nodule formation; fix, nitrogen fixation;

-, no growth, no nodulation or no nitrogen fixation
