# Supplementary material for: Draft genome sequence of type strain HBR26T and description of Rhizobium aethiopicum sp. nov
Source: Stand Genomic Sci. 2017 Jan 26;12:14. doi: 10.1186/s40793-017-0220-z (PMC5278577; doi:10.1186/s40793-017-0220-z)
Supplement: Additional file 2: Table S2. — Carbon sources utilization response between Rhizobium aethiopicum sp. nov. strains and Rhizobium etli CFN42T. (DOCX 25 kb) [file 40793_2017_220_MOESM2_ESM.docx]

**Additional file2 Table S2.** Carbon sources utilization response between *Rhizobium aethiopicum* sp. nov. strains and *Rhizobium etli* CFN42^T^

| Carbon soursce | HBR23 | HBR26^T^ | HBR31 | HBR3 | CFN42^T^ |
| --- | --- | --- | --- | --- | --- |
| Water | - | - | - | - | - |
| α-Cyclodextrin | - | - | - | - | - |
| Dextrin | + | + | + | + | + |
| Glycogen | + | + | + | + | + |
| Tween 40 | - | - | - | - | - |
| Tween 80 | - | - | - | - | - |
| N-Acetyl-DGalactosamine | - | - | - | - | - |
| N-Acetyl-DGlucosamine | + | - | + | + | - |
| Adonitol | + | + | + | + | + |
| L-Arabinose | + | + | + | + | + |
| D-Arabitol | + | + | + | + | + |
| D-Cellobiose | + | + | + | + | + |
| I-Erythritol | + | + | + | - | - |
| D-Fructose | + | + | + | + | + |
| L-Fucose | + | + | + | + | + |
| D-Galactose | + | + | + | + | + |
| Gentiobiose | - | - | - | - | - |
| α-D-Glucose | + | + | + | + | + |
| m-Inositol | + | - | + | - | - |
| α-D-Lactose | + | + | + | + | + |
| Lactulose | + | + | + | + | + |
| Maltose | + | + | + | + | + |
| D-Mannitol | + | + | + | + | + |
| D-Mannose | + | + | + | + | + |
| D-Melibiose | + | - | + | + | + |
| β-Methyl-D-Glucoside | - | + | + | + | + |
| D-Psicose | + | + | + | + | + |
| D-Raffinose | + | + | + | + | + |
| L-Rhamnose | + | + | + | + | + |
| D-Sorbitol | + | + | + | + | + |
| Sucrose | + | + | + | + | + |
| D-Trehalose | + | + | + | + | + |
| Turanose | + | + | + | + | + |
| Xylitol | + | + | + | + | + |
| Pyruvic Acid Methyl Ester | + | + | + | + | + |
| Succinic Acid Mono-Methyl-Ester | + | + | + | + | + |
| Acetic acid | - | - | - | - | + |
| Cis-Aconitic acid | - | + | + | - | + |
| Citric acid | - | - | + | - | + |
| Formic acid | - | + | + | - | - |
| D-Galactonic Acid Lactone | - | + | - | - | - |
| D-Galacturonic acid | - | - | - | - | - |
| D-Gluconic acid | - | - | - | - | - |
| D-Glucosaminic acid | - | - | - | - | - |
| D-Glucuronic acid | - | - | - | - | - |
| α-Hydroxybutyric acid | + | - | + | - | - |
| β-Hydroxybutyric acid | + | + | + | + | / |
| γ-Hydroxybutyric acid | + | + | + | - | + |
| p-Hydroxy Phenylacetic acid | - | - | - | - | - |
| Itaconic acid | + | + | + | - | - |
| α-Keto Butyric acid | + | - | + | - | - |
| α-Keto Glutaric acid | + | + | + | - | - |
| α-Keto Valeric acid | - | - | - | - | - |
| D,L-Lactic acid | + | + | + | + | + |
| Malonic acid | + | - | + | - | - |
| Propionic acid | - | - | - | - | - |
| Quinic acid | - | - | + | - | - |
| D-Saccharic acid | - | - | - | - | - |
| Sebacic acid | - | + | - | - | - |
| Succinic Acid | + | + | + | + | + |
| Bromosuccinic acid | + | + | + | + | + |
| Succinamic acid | + | + | + | - | + |
| Glucuronamide | - | - | - | - | - |
| L-Alaninamide | + | + | + | + | + |
| D-Alanine | + | + | + | + | + |
| L-Alanine | + | + | + | + | + |
| L-Alanyl-glycine | - | + | + | + | - |
| L-Asparagine | - | + | + | + | + |
| L-Aspartic Acid | - | + | + | + | - |
| L-Glutamic acid | + | - | + | + | - |
| Glycyl-L-Aspartic acid | - | - | + | - | - |
| Glycyl-L-Glutamic acid | + | + | + | - | + |
| L-Histidine | + | + | + | + | - |
| Hydroxy-L-Proline | + | + | + | + | - |
| L-Leucine | - | + | + | - | - |
| L-Ornithine | - | + | + | + | - |
| L-Phenylalanine | - | - | - | - | - |
| L-Proline | + | + | + | + | - |
| L-Pyroglutamic acid | - | - | - | - | - |
| D-Serine | - | - | - | - | - |
| L-Serine | - | + | + | - | - |
| L-Threonine | - | - | + | - | - |
| D,L-Carnitine | + | + | + | + | + |
| γ-Amino Butyric acid | + | + | + | - | / |
| Urocanic Acid | + | + | + | + | / |
| nosine | + | + | + | - | / |
| Uridine | + | + | + | + | / |
| Thymidine | - | + | + | + | - |
| Phenyethyl-amine  Putrescine  2-Aminoethanol | -  -  - | -  -  - | -  -  - | -  -  - | -  -  - |
| 2,3-Butanediol | - | + | + | - | - |
| Glycerol | + | + | + | + | - |
| D,L-α-Glycerol Phosphate | - | + | - | - | - |
| α-D-Glucose-1-Phosphate | - | + | + | + | - |
| D-Glucose-6-Phosphate | + | + | + | + | - |

+, growth; /, weak growth; -, now growth
